# The Role of the Gut–Liver Axis in the Development of Liver Diseases Induced by Selected Environmental Pollutants: A Narrative Review

**DOI:** 10.3390/ijms27198581

**Published:** 2026-09-25

**Authors:** Sara Jarmakiewicz-Czaja, Kübra Ergan, Aneta Sokal-Dembowska

**Affiliations:** 1 Faculty of Health Sciences and Psychology, Collegium Medicum, University of Rzeszow, 35-959 Rzeszow, Poland; sjczaja@ur.edu.pl; 2 University Center for Research and Development in Health Sciences, University of Rzeszow, 35-959 Rzeszow, Poland; 3 Department of Nutrition and Dietetics, Faculty of Health Sciences, Istanbul Yeni Yuzyil University, Istanbul 34010, Turkey; kubra.ergan@yeniyuzyil.edu.tr

**Keywords:** gut–liver axis, microbiota, environmental exposure, heavy metals, particulate matter, endocrine-disrupting chemicals, liver disease

## Abstract

Environmental pollution has emerged as a critical global health threat, with xenobiotic exposure increasingly linked to the pathogenesis of chronic liver diseases. The gut–liver axis has been proposed as a potential mediator in the body’s response to these insults. This review evaluates how selected environmental pollutants—heavy metals, particulate matter (PM), and endocrine-disrupting chemicals (EDCs), particularly Bisphenol A (BPA)—disrupt this homeostatic balance. Evidence indicates that environmental toxins induce gut dysbiosis and impair intestinal barrier integrity. This “leaky gut” phenomenon facilitates the translocation of endotoxins (LPS) into the portal circulation, where they trigger the TLR4/NF-κB signaling pathway in the liver. This leads to the activation of chronic inflammation, oxidative stress, and significant lipid accumulation in the liver. Furthermore, EDCs interfere with hepatic nuclear receptors, directly deregulating lipid metabolism and driving the progression of metabolic dysfunction-associated steatotic liver disease (MASLD). By highlighting the gut–liver axis as a key pathway affected by selected environmental contaminants, this review provides a mechanistic foundation to guide future research toward targeted preventive and therapeutic strategies.

## 1. Introduction

Exposure to various environmental pollutants is an important public health concern worldwide [1]. Heavy metals, particulate matter (PM), polycyclic aromatic hydrocarbons (PAHs), and endocrine-disrupting chemicals (EDCs) are ubiquitous in the environment, originating from widespread industrial emissions, vehicular exhaust, and contaminated food or water [2,3,4,5,6]. Chronic exposure to these contaminants is strongly linked to the rising global prevalence of metabolic dysfunction-associated steatotic liver disease (MASLD) [7], and recent studies suggest a potential contributing role of the gut microbiome and the gut–liver axis in mediating their harmful systemic effects [8,9,10].

Understanding the toxic effects of these contaminants requires first establishing baseline physiology. The gut–liver axis represents a bidirectional communication network established primarily through the portal circulation and the biliary system. Under normal physiological conditions, an intact intestinal barrier, comprising the mucus layer, epithelial cells sealed by tight junctions, and the gut vascular barrier, maintains selective permeability, allowing nutrient absorption while restricting the systemic dissemination of luminal microbes and endotoxins. The portal vein conveys gut-derived substances and microbial signals directly to the liver, which acts as a primary immunological and metabolic filter. A balanced gut microbiota further supports this homeostasis by modulating mucosal immunity and producing beneficial metabolites, such as short-chain fatty acids (SCFAs) [11].

Environmental pollutants are recognized as xenobiotics by the organism, directly disrupting the intestinal microbial balance. Such disruption can lead to a drastic decline in the number of beneficial bacteria and reduced species diversity [10,12,13,14] and the development of leaky gut syndrome [14,15,16].

The loss of the physical protective barrier initiates a series of adverse changes. Pro-inflammatory molecules and endotoxins, including lipopolysaccharides (LPS), enter the bloodstream and reach the liver directly via the portal vein [14,15,16,17,18,19]. This continuous influx of toxins from the intestinal environment triggers an inflammatory cascade, exacerbates oxidative stress and excessive lipid accumulation, paving the way for the development of chronic liver damage, including MASLD and fibrosis [17,19,20,21,22,23]. Understanding the pathological links within the gut–liver axis is therefore key to comprehending how daily exposure to environmental pollutants drives chronic disease processes. While recent reviews often address pollutant-induced gut dysbiosis or liver toxicity in isolation, there is still a gap in connecting these two processes. Therefore, this narrative review focuses specifically on selected environmental pollutants—namely, heavy metals, particulate matter, and selected EDCs. Our goal is to synthesize research findings, including those that analyze effects on the gut or liver separately, to highlight their impact on the gut–liver axis.

## 2. Methodology

To provide a comprehensive synthesis of the mechanistic interactions between environmental pollutants, gut homeostasis, and liver health, a literature search was conducted across the PubMed/MEDLINE, Scopus, Web of Science, and Google Scholar databases, with the final search completed in June 2026. The search covered peer-reviewed articles published in English between 2005 and 2026. The 2005 cutoff was chosen to capture the early foundational studies on gut microbiome toxicology, while the primary focus remained on the last decade (2016–2026) to reflect recent molecular advances. Study screening and selection were performed by three reviewers (S.J.-C., A.S.-D., and K.E.), with 128 articles ultimately selected and included in the final synthesis based on their direct relevance to the gut–liver axis. To avoid repeating the same underlying evidence from secondary sources, we prioritized primary original research.

Medical Subject Headings (MeSH) and free-text keywords were combined using logical Boolean operators (AND, OR). The search strategy structured terms into three principal domains:

- Environmental Toxicants and Pollutants: “heavy metals” (e.g., “lead”, “cadmium”, “mercury”, “arsenic”), “air pollution”, “particulate matter” (“PM2.5”, “PM10”), “polycyclic aromatic hydrocarbons” (“PAHs”), and “endocrine-disrupting chemicals” (“EDCs”, including “bisphenol A/BPA”, “BPF”, “BPP”, “dibutyl phthalate/DBP”, and “DEHP”).
- Gut Microbiota and Intestinal Barrier: “gut microbiota”, “gut dysbiosis”, “gut barrier”, “intestinal barrier”, “intestinal permeability”, “leaky gut”, and “short-chain fatty acids” (“SCFAs”).
- Liver Outcomes and Mechanistic Pathways: “gut–liver axis”, “metabolic dysfunction-associated steatotic liver disease” (“MASLD”), “non-alcoholic fatty liver disease” (“NAFLD”), “liver health”, “oxidative stress”, and “inflammation”.

### Inclusion and Exclusion Criteria

The evaluation included original research articles (preclinical in vitro and in vivo animal models, as well as human observational and comparative studies), systematic reviews, meta-analyses, and comprehensive narrative reviews published in English. Narrative reviews were considered for background and contextual information but were not included in the evidence synthesis. Literature search results across selected databases were screened, and duplicates were removed. The relevance and reliability of the included evidence were evaluated based on publication in peer-reviewed journals, study design rigor (e.g., appropriate control groups and clear mechanistic endpoints), and direct alignment with xenobiotic-induced gut–liver outcomes. Studies were selected if they directly investigated the mechanistic relationships linking environmental xenobiotic exposure to gut microbiota dysbiosis, gut barrier dysfunction, or hepatic injury. Studies were included if they evaluated pollutant-induced impacts on the gut–liver axis as a unified system, or if they assessed specific intestinal or hepatic outcomes to characterize individual components of the proposed axis rather than direct mediation. Articles unrelated to environmental exposure, conference proceedings, non-peer-reviewed literature, and articles with unavailable full texts were excluded.

## 3. Heavy Metals

Heavy metals are elements that cannot be broken down in nature and can bioaccumulate through the food chain, capable of having serious toxic effects on biological systems even at low concentrations [2]. The main heavy metals that pose the greatest threat to human health are lead, cadmium, mercury, and arsenic [3]. In this section, the term “heavy metals” is used as a broad concept encompassing toxic metals and metalloids; indeed, arsenic is technically a metalloid, while copper and zinc are essential trace elements that only become toxic at excessive exposure levels. When these elements enter biological systems, they are not metabolically degraded; however, they can undergo biotransformations such as methylation, oxidation state (redox) changes, binding to biological ligands, sequestration, and ultimately excretion. Their accumulation in tissues and the liver triggers pathological processes such as oxidative stress, inflammation, and organ damage [24].

### 3.1. Exposure Characteristics and Sources

This review focuses on cadmium, lead, mercury, and arsenic. This selection is based on the fact that these elements constitute the most common sources of general population exposure through the environment and food and that the strongest epidemiological and mechanistic evidence regarding their involvement in hepatotoxicity and gut–liver axis pathologies is available for these elements. The depth of the mechanistic discussion regarding these elements reflects the scope of the available evidence concerning the association of each element with the intestinal barrier and hepatic pathology. Exposure to heavy metals occurs through air, water, and food as a result of environmental pollution and industrial activities [7]. According to the type of exposure, the main sources can be categorized as follows:

- Cadmium

The primary sources of exposure for the general population are food and drinking water [25]. In smokers, tobacco smoke is the primary source; in non-smokers, cadmium enters the diet primarily through plants as a result of its accumulation in the soil [26]. Additionally, nickel–cadmium batteries, industrial paints, and fertilizers are among the common sources [27].

- Lead

Air, dust, soil, and lead-containing paints are major sources of exposure [28]. Leaking water from plumbing pipes in old buildings, industrial emissions, and some food containers can also cause lead buildup [29].

- Mercury

Humans are most commonly exposed to mercury through the consumption of seafood contaminated with methylmercury (especially tuna, shark, and swordfish) [30]. Other sources include dental amalgams, thermometers, and some disinfectants. Toxicity among mercury types varies significantly: methylmercury is a potent neurotoxicant that biomagnes in the food chain; inorganic mercury, on the other hand, primarily exhibits renal and gastrointestinal toxicity. Therefore, the chemical form of the element must be considered in exposure assessments [30,31].

- Arsenic

The primary route of exposure is drinking contaminated groundwater and consuming agricultural products irrigated with this water [32].

### 3.2. Effects of Heavy Metals on Gut Microbiota and the Gut Barrier

Human intestinal microbiota has become a focus of toxicological research in recent years due to its critical roles in host homeostasis, immune development, and metabolic processes [8]. Heavy metals, which are among environmental pollutants, are considered xenobiotics that disrupt microbiota composition and species diversity, triggering negative health consequences on host physiology [12]. Exposure to metals such as arsenic, cadmium, and nickel, in particular, causes dose-dependent changes in the intestinal flora, leading to dysbiosis [13]. Disruption of microbial balance is associated with conditions such as leaky gut syndrome, oxidative stress, and inflammatory bowel diseases [33]. Metal-induced dysbiosis alters the production of microbial metabolites such as short-chain fatty acids and bile acids [34]. These alterations may contribute to disturbances in host lipid and glucose metabolism [35]. Studies in animal models have shown that mercury exposure increases levels of Proteobacteria while decreasing the amounts of beneficial genera such as Bacillus [36,37].

However, these changes in microbiota composition are not only an indicator of pathogenicity but also have the potential to be a preclinical biomarker in determining exposure to certain heavy metals [38]. Chronic intake of metals such as cadmium and lead leads to specific taxonomic shifts in gut microbial communities, resulting in significant declines in populations of beneficial families such as Lachnospiraceae [39]. Furthermore, arsenic exposure has been observed to disrupt intestinal metabolic functions by suppressing butyrate-synthesizing bacterial populations and directly affect immune modulation processes [40].

Heavy metal-induced changes in the gut microbiota have systemic effects that extend beyond the digestive system [41]. Changes in microbial composition are associated with increased glucose levels, body weight changes, and neurological dysfunctions [42,43]. The relationship between microbiota and metals is bidirectional. Microbiota can be affected by heavy metals, but it can also modulate their bioavailability and toxicity. In particular, some beneficial bacteria, such as *Lactobacillus* species, can bind metals, reducing their absorption from the gut [44].

The presence of heavy metals adversely affects the composition of gut microbiota, leading to a deterioration of intestinal barrier integrity and immune homeostasis via mechanisms such as redox imbalance and the activation of Toll-like receptor 4 (TLR4)/ nuclear factor kappa B (NF-κB) and NOD-like receptor family pyrin domain-containing 3 (NLRP3) signaling pathway [44]. Heavy metals, particularly cadmium, disrupt tight junctions between epithelial cells by altering the expression of proteins such as zonula occludens-1, occludin, and claudin, thus contributing to the development of leaky gut syndrome [45]. Cadmium exposure in particular can completely impair barrier function by reducing transepithelial electrical resistance [46]. Heavy metals have been shown to induce epithelial cell death by causing oxidative stress and an increase in reactive oxygen species [47]. Disruption of barrier integrity allows pro-inflammatory molecules, such as lipopolysaccharides, to enter the bloodstream and reach the liver via the portal vein. This process exacerbates hepatic inflammation and liver damage via the gut–liver axis [17]. Collectively, these findings suggest that heavy metal-induced disruption of epithelial integrity may represent a central mechanistic link connecting environmental exposure to chronic hepatic inflammation and the progression of MASLD.

The presence of heavy metals in the intestinal microenvironment weakens the host’s natural detoxification capacity by leading to the suppression of specific bacterial species and a decrease in microbial diversity [48]. Specifically, this decrease in the population of butyrate-producing Gram-positive bacteria leads to a restriction in short-chain fatty acid production and a consequent increase in intestinal pH, making epithelial integrity more vulnerable [49]. This situation facilitates the passage of bacterial translocation and toxic metabolites into the portal circulation by disrupting the selective permeability of the intestinal barrier, triggering inflammatory responses in the liver and deepening hepatic damage [50]. Deterioration of intestinal barrier integrity triggers a positive feedback mechanism that increases cadmium and arsenic absorption, accelerating the metal’s accumulation in hepatic tissues and reinforcing the toxicity cycle [51]. Similarly, lead exposure increases intestinal permeability, facilitating the entry of inflammatory cytokines and microbial metabolites into the enterohepatic circulation, thus contributing to the development of multiple systemic lesions. In this context, nutritional interventions such as dietary fiber, probiotic, and vitamin C supplementation stand out as a potential protective strategy in mitigating lead toxicity by maintaining intestinal integrity. It is thought that lead exposure increases intestinal permeability, facilitating the entry of inflammatory cytokines and microbial metabolites into the enterohepatic circulation, thus contributing to the development of multiple systemic lesions [52]. Similarly, mercury alters the gut microbiota composition in animal models [36,37], is identified as the most potent hepatotoxin in human cohorts, and combined exposure to metals is reported to exacerbate liver damage [53].

Heavy metal exposure has also been associated with impaired goblet cell function and reduced mucus production. A healthy mucus layer contributes to the protection of the intestinal barrier by binding to metals and facilitating their removal, while damage to the mucus layer weakens this defense mechanism and can increase susceptibility to metal toxicity [54].

### 3.3. Consequences for Liver Function

The effects of heavy metals on liver function are extensively discussed in the literature, both through direct hepatotoxic mechanisms and indirect processes occurring via the gut–liver axis. Metals such as lead, cadmium, mercury, and arsenic are not biodegradable and tend to accumulate in the liver [55]. Heavy metal accumulation leads to excessive production of reactive oxygen species, oxidative stress, and lipid peroxidation, triggering hepatocellular necrosis [19].

Recent studies have shown that heavy metals disrupt the composition of the gut microbiota, leading to dysbiosis and weakening the integrity of the intestinal barrier [55]. Increased intestinal permeability accelerates the passage of endotoxins, such as lipopolysaccharides, into the liver, exacerbating liver inflammation, steatosis, fatty liver disease, and even fibrosis [18]. Human studies have shown that exposure to heavy metal mixtures significantly increases the risk, particularly of MASLD [20,56]. In addition, it has been reported that high nickel and copper levels are associated with gastrointestinal complaints such as upper gastrointestinal bleeding, abdominal pain, burning sensation and diarrhea; and high manganese (2.11 ppm), lead and nickel levels are identified as the source or trigger of chronic liver disease [57].

The effects of heavy metals on humans and animals are presented in Table 1.

Much of the mechanistic evidence regarding the effects of heavy metals on gut microbiota and barrier function has come from animal models; for example, the study by Breton et al. used high and heterogeneous dose regimens such as cadmium 20–100 ppm and lead 100–500 ppm [39]. These doses may not fully reflect real-life exposure levels. Human data, on the other hand, are predominantly based on cross-sectional cohort studies, and longitudinal follow-up data are limited; this also restricts the establishment of causal relationships between exposure and hepatic outcomes. Furthermore, gender differences have not been examined in detail in most of the existing studies, and methodological heterogeneity in microbiome analyses makes inter-study comparisons difficult. Since confounding factors such as diet and geographic region can affect both metal exposure and microbiota composition, the generalizability of the findings is limited. On the other hand, in real life, individuals are exposed not to a single metal, but to mixtures of metals and other pollutants. Future studies should conduct long-term prospective designs examining low doses and realistic mixed exposures, using standardized microbiome analysis methods and controlling for gender differences and dietary/geographical factors, to elucidate causal relationships.

## 4. Air Pollution (PM2.5/PM10 and PAHs)

### 4.1. Characteristics and Routes of Exposure

Particulate matter (PM) is a significant air pollutant. Depending on particle diameter, it is classified into, among others, coarse particles (PM with an aerodynamic diameter of less than 10 μm, PM10) and fine particles (PM with an aerodynamic diameter of less than 2.5 μm, PM 2.5), which serve, for example, as air quality indicators and are widely used in health risk assessments. PM can be emitted directly, such as from engine exhaust or volcanic ash, or formed secondarily in the atmosphere as a result of chemical reactions [6,60]. The toxicity of PM depends not only on particle size but also on their chemical composition [61]. However, further research is needed to determine the differences in toxicity among the individual components of PM [62].

PAHs are compounds with at least two aromatic rings. They are produced by natural emission sources, such as forest fires, or by anthropogenic emission sources, such as mobile, industrial, and residential sources [5,63]. Due to their hydrophobic properties, PAHs easily adsorb onto the surface of fine PM2.5 particles, which facilitates their transport deep into the respiratory system. Benzo[a]pyrene is of particular toxicological significance. It is considered an indicator of environmental PAH contamination and a carcinogenic compound. It can be found in soil, air, water, food, etc. [64]. PAHs can be classified into those with low molecular weight, MW < 200 g/mol; those with medium molecular weight, 200 ≤ MW < 250 g/mol; and those with high molecular weight, MW ≥ 250 g/mol [6].

The PM2.5 fraction may contain approximately 65-95% of all PM-associated PAHs, indicating that PAHs are primarily found in fine PM [6]. In addition, meteorological factors such as temperature, wind, and humidity influenced concentrations by altering the gas-particle partitioning, dispersion, and removal from the atmosphere [65]. Higher concentrations were also observed in industrial and port areas than in urban areas [65,66].

Exposure to PM and PAHs occurs primarily through inhalation, although exposure via the digestive tract and skin contact are also significant. The extent of exposure depends, among other factors, on the concentration of pollutants in the environment and the duration of exposure to polluted air. Larger particles are deposited mainly in the nasal cavity, throat, and larynx, while finer particles settle in the bronchioles and alveoli. Due to their small size, they can cross the alveolar–capillary barrier and enter the systemic circulation [67,68]. The second main route of exposure is through the digestive tract or the skin [69,70]. PAHs can penetrate the stratum corneum and be absorbed into the organism [71]. Once PM enters the human body, it affects various systems, organs, and the gut microbiota.

Inhaled PM can reach the gastrointestinal tract as a result of mucociliary clearance in the respiratory tract, after which they are swallowed. In the case of PAHs associated with PM, the inhalation route may be of particular importance, as these compounds can partially bypass first-pass metabolism in the liver, facilitating their further distribution throughout the body. In contrast, PAHs ingested with food are absorbed in the gastrointestinal tract and then transported to the liver, where they undergo first-pass metabolism [72,73,74].

### 4.2. The Effect of PM and PAHs Exposure on the Gut Microbiota and the Gut Barrier

Air pollutants, including PAHs, may also affect the composition and functions of the gut microbiota as well as the maintenance of intestinal barrier homeostasis [9]. In their study using animal models, Xie et al. observed that PM2.5 damaged the mucus layer and altered the microbiota depending on the dose and duration of exposure. After 24 weeks of exposure to concentrated ambient PM2.5, Proteobacteria levels were significantly lower [75]. In human studies, no consistent taxonomic changes were reported. However, shifts in Bacteroidetes and Proteobacteria, among others, were the most frequently observed [14].

Importantly, PM should not be treated as a homogeneous exposure factor, as its biological effects may vary depending on particle size and chemical composition. In particular, PM_2.5_ and PM_10_ differ in terms of their physicochemical properties, deposition patterns, and associated chemical constituents [76]. In their systematic review of 48 studies, Van Pee et al. identified an association between higher PM exposure and lower gut microbiota diversity. However, they note that further human studies are needed to confirm these associations and elucidate the underlying mechanisms [14]. In turn, studies using animal models have shown that exposure to PM can cause changes in the structure of the gut microbiota. One example is PM inhalation, which altered the microbiota throughout the entire gastrointestinal tract in mice. However, researchers observed the greatest changes in the distal sections of the intestine. Changes were primarily noted in the beta-diversity of the gut microbiota [77]. Available data indicate that exposure to PM may affect the diversity of the gut microbiota; however, the direction of these changes may depend on the particle fraction, exposure dose, duration of exposure, and experimental model. PM_2.5_ particles have been studied in greater detail than PM_10_ particles with regard to changes in the gut microbiota, while data on the specific effects of PM_10_ particles remain limited [78].

Furthermore, changes in the diversity of the gut microbiota are associated with the metabolites it produces. These changes can lead to reduced production of butyrate and other key metabolites responsible for maintaining the integrity of the intestinal barrier and the proper regulation of the immune response. Additionally, the activation of the NLRP3 and TLR4/NF-κB pathways has been mechanistically implicated [44,79]. Studies have also been conducted on the effects of PAHs on the gut microbiota. Jeong et al. indicate that exposure to PAHs may cause a decrease in Firmicutes and an increase in Bacteroidetes [80]. In another study using animal models, Ribiere et al. observed that oral exposure to benzo[a]pyrene caused changes in both the composition and abundance of the gut microbiota. Furthermore, it induced inflammation in the intestinal mucosa [81]. Following exposure to PAHs from PM10, the diversity of the gut microbiota returned to normal, but inflammation persisted, which may predispose individuals to the development of inflammatory bowel disease [80].

The effect of PM on the intestinal barrier has also been demonstrated. Exposure to PM may reduce the integrity of the intestinal barrier by damaging the mucus layer and disrupting the expression of tight junction proteins (TJP), such as zonula occludens-1 (ZO-1) and claudin-1, which may lead to increased intestinal epithelial permeability. This, in turn, predisposes the system to the translocation of, for example, bacterial LPS into the circulation, as well as increased bacterial translocation, thereby initiating chronic inflammation [14]. In their study using animal models, Ran et al. observed that chronic exposure to PM2.5 can lead to disturbances in the homeostasis of the intestinal barrier. Additionally, it may be associated with changes in intestinal metabolites and the activation of the TLR2/5-MyD88-NLRP3 inflammasome in intestinal dysfunction [82]. Furthermore, Mutlu et al. indicated that exposure to PM can cause the apoptosis of epithelial cells in the gastrointestinal tract. Moreover, due to its adverse effect on TJP, intestinal permeability increases and inflammation is exacerbated. The authors note that chronic exposure to PM may lead to exacerbation of gastrointestinal inflammation [83]. In another study, Liao et al. demonstrated in a cellular model that PI3K/AKT-dependent production of reactive oxygen species (ROS) following exposure to PM2.5 can lead to impaired intestinal barrier function [84]. In turn, Fitch et al., while studying the effects of PM on animal models, found reduced Muc2 expression, which in turn may weaken the protective intestinal mucosal barrier and contribute to increased intestinal permeability [85]. In a study by Gao et al., the authors observed that exposure to PM2.5 disrupts the function of mucus-producing goblet cells in the intestines, which may exacerbate intestinal barrier dysfunction [86]. Xie et al. also confirmed that exposure to PM2.5 can alter the production of intestinal metabolites, which may serve as a nutrient source for enterocytes, thereby predisposing them to intestinal barrier impairment. Additionally, they indicate that gut dysbiosis may be a precursor to the development of certain metabolic diseases [75].

Exposure to PAHs may be associated with damage to the intestinal epithelium, mucosal inflammation, and impaired intestinal barrier function [87]. Huang et al. conducted a similar study assessing the association between exposure to electronic waste and the prevalence of inflammation in children. They observed reduced levels of SIRT6 (sirtuin 6) in children with impaired intestinal barrier function. Additionally, they found correlations between SIRT6 levels and PAHs concentrations [88]. Kakutani et al. observed that persistent organic pollutants (POPs), including PAHs, exhibit the ability to bind to aromatic hydrocarbon receptor, which, by altering the TJP, can disrupt the homeostasis of the intestinal barrier [89]. PAH molecules can selectively cross the intestinal barrier depending on their physicochemical properties. Furthermore, Caco-2 cells metabolize PAHs, which may increase their transfer through the epithelium. Intestinal metabolism may influence the bioavailability of PAHs in the organism [90]. In adults, higher exposure to PAHs may predispose individuals to chronic diarrhea. However, no such correlations have been observed in the case of constipation [91].

Air pollution, particularly PM_2.5_, can disrupt the composition and diversity of the gut microbiota, reduce the production of beneficial metabolites, and activate pro-inflammatory pathways. PM particles can also damage the intestinal barrier and disrupt its homeostasis, which may lead to increased intestinal permeability. However, the strongest scientific evidence pertains to PM2.5 particles, while data on polycyclic aromatic hydrocarbons (PAHs) remain limited.

### 4.3. Liver Consequences of PM Exposure

PM particles affect not only the gut microbiota and the intestinal barrier but also, through their effects, many organs, including the liver. Exposure to PM2.5 is primarily associated with an increased risk of fatty liver disease in humans, with the risk being higher among overweight individuals and smokers. In contrast, animal models have shown that PM2.5 also influences the development of inflammation and fibrosis in the liver [21]. In their meta-analysis, Pan et al. report that exposure to PM2.5 leads to elevated levels of liver enzymes (gamma-glutamyl transferase (GGT), alanine aminotransferase (ALT), and aspartate aminotransferase (AST)), causing liver damage [92]. In another study, Zheng et al. indicate that exposure to PM2.5 induces non-alcoholic steatohepatitis (NASH)-like changes in the liver and may also disrupt glucose metabolism [93]. NADPH oxidase probably has a significant effect on fibrinogenesis in the liver following exposure to PM2.5 [94]. Exposure to PM2.5 may activate the AMPK-SIRT-CerS6 axis through elevated cortisol levels, which predisposes the organism to mitochondrial damage and the accumulation of ceramide lipids, thereby increasing the risk of hepatocellular carcinoma [95]. In their meta-analysis of 16 studies, Siu et al. observed that individuals exposed to PM2.5 concentrations 10 μg/m^3^ higher had an approximately 27% greater risk of developing chronic liver disease compared to those exposed to lower concentrations [96]. The main mechanisms involve the activation of pathways mediated by TLR4, c-Jun N-terminal kinase (JNK), and nuclear factor kappa B (NF-κB) [93]. Additionally, PM2.5 may influence both the inflammatory response of hepatocytes and lipid metabolism disorders, as well as the activation of hepatic stellate cells [21]. In another study, Yan et al. indicate that exposure to PM2.5 may cause iron accumulation and activate ferritinophagy through NCOA4 [97]. Furthermore, hepatocyte toxicity was caused, among other things, by the activation of pyroptosis and ferroptosis [97,98]. Additional factors may also influence liver condition following PM exposure. For example, it has been shown that a high-fat diet and exposure to PM2.5 particles interact synergistically, exacerbating oxidative stress and the inflammatory response [99]. Furthermore, the composition of PM and its content of metals, as well as soluble and insoluble fractions, may affect liver health [100,101]. PM can affect the liver in multiple ways, ranging from impaired liver enzyme function, through effects on lipid and glucose metabolism, to an increased predisposition to liver fibrosis. However, the final effect depends on the type of PM, the frequency of exposure, and other predisposing factors such as diet.

However, the available evidence is heterogeneous, as the hepatic effects of PM_2.5_ may depend on particle composition, exposure frequency, and individual or environmental factors such as diet. Further studies are needed to clarify the contribution of specific PM components and the underlying mechanisms linking PM exposure with liver injury, fibrosis, and metabolic disturbances.

Table 2 summarizes the effects of PM and PAHs on the intestines and liver.

## 5. Selected Endocrine-Disrupting Chemicals (Bisphenols and Phthalates) and the Gut–Liver Axis

Given the vast diversity of EDCs, a comprehensive analysis of all classes is beyond the scope of this work. Therefore, this section explicitly focuses on selected bisphenols (including BPA and its analogs like BPF and BPP) and phthalates (such as DBP and DEHP), as they are currently the most extensively documented in relation to gut–liver axis pathogenesis. EDCs can interfere with the way many of the body’s systems function. Their negative effects on the reproductive system, the cardiovascular system, the nervous system, and the brain have been documented. Furthermore, they may increase the risk of cancer and affect the health of future generations [4]. Exposure to EDCs during pregnancy can lead to epigenetic changes in the germline and disrupt the programming of somatic tissues. These changes can be passed on to subsequent generations and affect, among other things, the onset of puberty and the development of the endocrine system [104]. However, as Chen et al. emphasize, much of the scientific evidence comes from observational studies, so it is difficult to establish a cause-and-effect relationship between exposure and the risk of developing these disorders. Additionally, these studies are often characterized by high heterogeneity, resulting from differences in the methodologies used [105].

In recent years, the role of the gut microbiota in mediating the development of various diseases has also been emphasized. However, few studies have examined the relationship between EDCs and the gut microbiota [10]. Gut dysbiosis often accompanies liver disease. In patients with non-alcoholic fatty liver disease (NAFLD)/MASLD and liver cirrhosis, reduced indicators of microbiota diversity (Shannon and Chao1 indices) and characteristic changes in its composition have been observed [106,107]. Clinical studies indicate that interventions targeting changes in the gut microbiota can lower levels of inflammatory markers, improve metabolic parameters and reduce the extent of liver damage [108,109].

### 5.1. Characteristics and Sources of Exposure 

It is estimated that there are at least 1000 substances that can potentially disrupt the endocrine system [110]. EDCs can interfere with endocrine function through various mechanisms. They can both inhibit and stimulate hormone synthesis. Furthermore, they can affect the signaling of nuclear and non-steroidal receptors, as well as influence transcriptional coactivators and enzymes involved in the biosynthesis and metabolism of steroid hormones [111].

EDCs can enter the body through multiple routes, increasing the risk of their accumulation. EDCs are classified as either naturally occurring or synthetic [112]. The main classes of EDC include bisphenols (BPs), phthalates, perfluoroalkyl substances (PFAS), pesticides, polychlorinated biphenyls (PCBs), polybrominated diphenyl ethers (PBDEs) and parabens [113].

Table 3 below shows the acceptable daily intake limits for the compounds discussed in this section of the article.

Natural EDCs, on the other hand, mainly comprise phytoestrogens obtained through diet [10]. EDCs may be present in food because these substances can migrate from packaging produced using synthetic EDCs, or they can accumulate during crop cultivation. They can also enter the body through contaminated water. EDCs are also absorbed into the body through the air and skin contact, for example when using personal care products [112]. Consequently, they pose a real risk to humans and animals [113]. Recently, the impact of environmental pollutants on the development of gut dysbiosis and metabolic dysfunction has become a key area of research [119].

### 5.2. Effects of EDCs on the Gut Microbiota and the Gut Barrier

The gut microbiota is now recognized as an autonomous endocrine organ, making it a direct target of EDCs. While the exact molecular mechanisms of these interactions are not fully understood, exposure to EDCs has been shown to induce dysbiosis. This is characterized by a shift in the Bacteroidetes to Firmicutes ratio and a significant decline in the abundance of beneficial bacteria belonging to genera such as *Bifidobacterium* and *Lactobacillus* [10,120]. Experimental studies (in vivo and in vitro) show that EDCs can reduce the expression of tight junctions and thereby increase gut permeability [10]. For instance, California mice were exposed to bisphenol A (BPA and ethinyl estradiol (EE) during the perinatal period. It was demonstrated that these compounds alter the gut microbiome in a sex- and generation-dependent manner. In the group exposed to BPA, a significant decrease in bacteria of the genus *Lactococcus*, which are responsible for lactic acid production and protection of the intestinal barrier, was observed. This was accompanied by an increase in bacteria associated with the development of inflammatory bowel disease (IBD), colorectal cancer, and metabolic disorders [121]. These observations are confirmed by studies involving humans. In an observational study by Linares et al., which involved 200 patients (60 of whom had active disease), high levels of BPA were observed. These levels correlated with both disease activity and phenotype. Patients with higher levels of this EDC had higher levels of circulating bacterial DNA and LPS. Furthermore, this group exhibited lower serum concentrations of SCFAs and reduced serum tryptophan levels [122]. These findings are consistent with the analysis by Wu et al. on the effects of EDCs on the gut microbiota in the context of attention-deficit/hyperactivity disorder (ADHD) [120].

Despite growing clinical and experimental evidence linking EDC exposure to gut dysbiosis, further research is needed to fully elucidate the underlying molecular pathways.

### 5.3. Liver Consequences of EDCs Exposure

A growing body of evidence suggests an association between EDC exposure and adverse liver outcomes [22,23,123]. A systematic review of studies conducted on the National Health and Nutrition Examination Survey (NHANES) and Korean National Environmental Health Survey (KoNEHS) cohorts identified an association between liver disease prevalence and exposure to certain phthalates [22]. However, it should be emphasized that most of the data currently available on the effects of EDCs on liver health via the gut–liver axis come from preclinical studies. Zhao et al. presented similar findings, noting an association between the presence of specific phthalate breakdown products in urine and an increased risk of MASLD progression. They also indicated that higher serum BPA levels promote the active phase of metabolic diseases and inflammation [123]. However, these analyses were limited by the partial overlap of the study populations [22] and their cross-sectional nature. Therefore, future longitudinal studies are needed that take repeated measurements of phthalate and BPA concentrations while monitoring long-term changes in the liver [22,123].

In the study by Feng et al. [16], BPA was administered at 50 μg/kg bw/day, corresponding to the historical US EPA reference dose (RfD) established in 1988 [115]. Notably, this dose is substantially higher than the current EFSA TDI of 0.2 ng/kg bw/day established in 2023 [114]. Exposure to BPA resulted in gut dysbiosis and damage to the epithelial barrier, allowing endotoxins (LPS) to enter the bloodstream. This activated inflammatory pathways, leading to excessive lipid accumulation and the development of fatty liver disease [16]. In turn, Liu et al. noted similar disturbances in the microbiota and a decrease in the levels of SCFAs. Regarding the liver itself, severe oxidative damage stimulated programmed cell death (apoptosis) via the mitochondrial pathway [124]. Harmful effects have also been described for other bisphenols. Exposure to bisphenol F (BPF) at higher doses (5 and 50 μg/L) induced liver fibrosis, oxidative stress and significant disturbances in lipid metabolism. In contrast, a dose of 0.5 μg/L corresponds to BPF concentrations actually detected in the environment (e.g., in river water) [125]. Similarly, an increase in intestinal barrier permeability contributed to elevated LPS concentrations in the blood in the case of bisphenol P (BPP). Following TLR4 receptor activation, an inflammatory cascade (increased pro-inflammatory cytokines) and liver fibrosis were triggered. Importantly, antibiotic therapy (the ABX+BPP group) completely reversed the steatosis, inflammation and oxidative stress induced by this compound [126]. A similar mechanism has also been described in the context of exposure to dibutyl phthalate (DBP). DBP significantly reduced the expression of the peroxisome proliferator-activated receptor gamma (PPARγ) receptor, which plays a key role in normal lipid metabolism and the suppression of inflammation. LPS originating in the gut activates the TLR4 receptor protein in the liver. This stimulates the NF-κB transcription factor, leading to an inflammatory cascade and increased production of pro-inflammatory cytokines [127]. Short-term exposure to DEHP (di(2-ethylhexyl) phthalate) can also disrupt the gut–liver axis. This compound negatively affects the microbiota and intestinal metabolism, compromises the integrity of the intestinal barrier and alters gene expression in the liver. Consequently, it contributes to liver damage [128].

An analysis of preclinical studies suggests that exposure to EDCs may affect the integrity of the gut microbiota. This can lead to a decrease in the expression of TJP and a reduction in mucosal thickness [126]. Furthermore, animal models demonstrate that exposure to EDCs influences the development of dysbiosis. Changes within the gut can lead to inflammation, steatosis and impaired liver function, and may contribute to liver fibrosis. A detailed analysis of the above-mentioned preclinical studies is presented in Table 4. Available findings support a possible contribution of the gut–liver axis to EDC-induced hepatotoxicity, although further studies are required to establish direct causal mediation.

Nevertheless, the studies in question have certain limitations. For example, some of the doses used pose a risk of acute toxicity [124,128] rather than environmental toxicity which limits the applicability of the results to human risk assessment. Using only male mice [16,124,126,127,128] or failing to conduct a comparative analysis between the sexes [124] also constitutes a significant limitation, given that the response to EDCs may vary by sex due to differences in hormonal and metabolic conditions.

Contextualizing the experimental exposure doses (Table 4) with safety reference values (Table 3) reveals a clear divide between environmentally relevant and high-dose toxicological studies. While the dose of 50 μg/kg bw/day for BPA used by Feng et al. [16] represents the historical US EPA RfD, it is worth noting that this dose is now 250,000-fold higher than the 2023 EFSA TDI of 0.2 ng/kg bw/day [114]. For BPF, the lowest dosage (0.5 μg/L) directly aligns with reported environmental aquatic concentrations [125], whereas phthalate studies typically utilize doses exceeding human safety reference values. Specifically, DBP exposures of 0.1 and 1 mg/kg bw/day represent 10- and 100-fold exceedances, respectively, of the EFSA group TDI of 50 μg/kg bw/day [118,127], and the subacute DEHP dose of 3 g/kg bw/day constitutes an extreme toxicological model (60,000-fold higher than the TDI) rather than a realistic human exposure scenario [128].

## 6. Conclusions

Exposure to heavy metals, air pollutants such as PM and PAHs, and selected EDCs, including bisphenols and phthalates, may negatively affect the gut–liver axis. These compounds can disrupt the gut microbiome, contributing to dysbiosis, characterized by alterations in the abundance of specific microbial taxa and reduced microbial diversity. These disturbances, together with damage to the intestinal barrier and altered expression of key TJP, including ZO-1, occludin, and claudin-1, may increase intestinal permeability.

Increased intestinal permeability may facilitate the systemic translocation of endotoxins, particularly LPS, and pro-inflammatory mediators into the bloodstream and their transport to the liver via the portal circulation. In the liver, these microbial products and inflammatory signals can contribute to inflammatory responses, oxidative stress, and disturbances in lipid metabolism. Collectively, these processes may contribute to the development and progression of MASLD and, potentially, subsequent liver fibrosis following chronic exposure to environmental pollutants.

The studies discussed suggest that the gut–liver axis may act as a mediator of hepatotoxicity, particularly for specific pollutants (such as BPA and lead) for which direct mechanistic pathways have been most extensively investigated. However, direct causal evidence in humans remains limited, and the reliance on high-dose experimental models in the current literature warrants caution when extrapolating these findings to real-world human environmental exposure. A better understanding of the molecular mechanisms underlying these interactions may provide new opportunities for future prevention and therapeutic research. Ultimately, long-term prospective cohort and clinical studies are still required to validate these experimental mechanisms and determine the exact translation of these pathways to human health.

### Limitations and Future Perspectives

The main limitation of this analysis is its reliance primarily on preclinical animal models exposed to high, heterogeneous doses, which do not reflect real-world, chronic, low-level human exposure. Furthermore, the lack of long-term human studies, combined with significant confounding factors related to diet, geographic location, and sex, limits our ability to establish direct cause-and-effect relationships. The focus on individual contaminants rather than chemical mixtures further complicates the translation of these findings to human health.

Although current research has elucidated key pathways involved in environmental contaminant-induced liver injury, several areas require further investigation to address these limitations. First, future studies should investigate the synergistic or antagonistic effects of combined exposure to multiple contaminants, reflecting real-world co-exposure scenarios. Second, the identification of noninvasive biomarkers of contaminant exposure in feces or serum is essential for early detection and risk assessment. Third, the development of targeted intervention strategies, such as probiotics, prebiotics, and personalized dietary interventions to modulate the gut–liver axis, holds significant therapeutic potential. Finally, long-term human studies are needed to validate findings from animal models and determine whether the proposed mechanisms are relevant to human health.

## Figures and Tables

**Table 1:** Effect of heavy metal on the gut and the liver—data from animal and human studies.

Reference (Year)	Experimental Model/Study Design	Exposure	Gut Outcomes	Liver Outcomes	Evidence Linking Gut and Liver
	Human Studies on Heavy Metals				
Adetunji (2024) [58]	NHANES cohort;	Pb, Hg, Cd, and essential elements;	heavy metals compete with essential elements, disrupting intestinal absorption processes;	elevated levels of ALT, AST, ALP, and GGT enzymes and an imbalance between toxic metals and essential elements, have been associated with increased liver damage;	concurrent gut and liver findings
Li et al. (2023) [20]	6520 adults;	blood exposure levels to Cd, Pb, Hg, and Se;	not assessed	strong positive correlation between blood Cd and lead levels and MASLD;	single-component evidence
Shao & Zhu (2020) [59]	mining area residents (40 adults);	long-term environmental exposure to As, Cd, Cu, Pb, and Zn via contaminated crops and locally produced foodstuffs in a Pb–Zn mining and smelting area	increase in Proteobacteria and Bacteroidetes; decrease in *Actinobacteria* levels; significant decline in microbial diversity;	not assessed	single-component evidence
Zuo et al. (2026) [53]	58,864 German outpatients (data from 2014 to 2022);	blood levels of As, Pb, and Hg at non-occupational exposure levels;	not assessed	increased ALT and AST enzymes (hepatocellular damage) and changes in ALP levels (increase with Pb, decrease with As and Hg); mercury was identified as the most potent hepatotoxin, and combined exposure to metals exacerbated liver damage;;	single-component evidence
Muhammad et al. (2018) [57]	60 patients; randomly selected patients suffering from liver, kidney, and heart diseases;	Cu, Pb, Mn, and Ni; the levels of these metals in blood were detected at approximately 1000 times the standard permitted limits (ppm);	upper GI bleeding, stomach pain, burning sensation, abdominal pain, and diarrhea (particularly associated with high nickel and copper levels);	high levels of manganese (2.11 ppm), lead, and nickel have been identified as either a source or trigger for chronic liver disease;	concurrent gut and liver findings
Animal Studies on Heavy Metals					
Breton et al. [39]	25 BALB/c female mice (6 weeks old);	Cd (20 or 100 ppm) or Pb (100 or 500 ppm); continuous oral intake via drinking water for 8 weeks;	alterations in the gut microbiota; decreased Lachnospiraceae, increased Lactobacillaceae and Erysipelotrichaceae (including *Turicibacter* spp.). disruption of gut homeostasis;	the metal doses used in the study were reported to be unrelated to hepatotoxicity and did not cause a change in liver weight; however, previous reports have indicated that this exposure can lead to iron loss in tissues;	concurrent gut and liver findings

NHANES—national health and nutrition examination survey; Pb—lead; Hg—mercury; Cd—cadmium; ALT—alanine aminotransferase; AST—aspartate aminotransferase; ALP—alkaline phosphatase; GGT—gamma-glutamyl transferase; Se—selenium; MASLD—metabolic dysfunction-associated steatotic liver disease; As—arsenic; Cu—copper; Zn—zinc; Mn—manganese; Ni—nickel; GI—gastrointestinal; ppm—parts per million.

**Table 2:** Effects of PM and PAHs on the gut and the liver—data from animal and human studies.

Reference (Year)	Experimental Model/Study Design	Exposure	Gut Outcomes	**Liver Outcomes**	**Evidence Linking Gut and Liver**
Zhao et al. (2025) [102]	umbrella review—general human population (observational studies, including cohort, case–control, and cross-sectional studies); 43 primary studies included in 9 systematic reviews and meta-analyses;	mainly PM2.5	PM2.5 was linked to colorectal cancer risk	PM2.5 was associated with liver dysfunction, chronic liver disease, and liver cancer;	concurrent gut and liver findings
Yao et al. (2026) [103]	3671 Chinese adults aged 45 or older;	PAHs	increase PAHs was associated with higher odds of non-neoplastic gastrointestinal disease	not assessed	single-component evidence
Jeong (2025) [80]	female C57BL/6 mice (8 weeks old); animals randomly assigned to experimental and control groups *(n* = 10 per group);	polycyclic aromatic hydrocarbons extracted from PM10 (PM10-PAHs); doses of 50, 100, or 200 mg/kg body weight; daily oral exposure (gavage) for 1, 2, or 4 weeks;	inflammation of the large intestine, disturbances in the gut microbiota, damage to the intestinal barrier.	not assessed	single-component evidence
Xie (2022) [75]	30 male C57BL/6J mice (3 weeks old); animals randomly assigned to 6 groups (*n* = 5 per group) after a 1-week acclimatization period;	concentrated ambient PM2.5 (CAPM); mean concentrations of approximately 124.79, 83.77, and 68.55 μg/m^3^ compared with the control group (filtered air—12.85 μg/m^3^); whole-body inhalation exposure for 8, 16, or 24 weeks (8 h/day, 6 days/week);	changes in the composition of the gut microbiota, damage to the intestinal barrier, and intestinal inflammation.	not assessed	single-component evidence
Zheng et al. (2013) [93]	male C57BL/6 mice; animals assigned to experimental and control groups (filtered air—FA);	concentrated PM2.5; average concentration approximately 10-fold higher than ambient levels (≈74.6 μg/m^3^ in the chamber vs. 6.5 μg/m^3^ outdoors) compared with the control group; whole-body inhalation exposure for 3 or 10 weeks (6 h/day, 5 days/week);	not assessed	NASH-like changes in the liver;	single-component evidence
Pan et al. (2023) [92]	more than 14 million human participants; participants included in 10 observational epidemiological studies (including cohort, cross-sectional, and longitudinal studies);	PM2.5; exposure increase analyzed per 10 μg/m^3^ increment; variable long-term and short-term environmental exposure to ambient air pollution;	not assessed	elevated liver enzyme levels;	single-component evidence
Yan et al. (2026) [97]	40 male C57BL/6J mice (6 weeks old); animals randomly assigned to four groups (PM2.5, DFO, PM2.5 + DFO, and control groups) (*n* = 10 per group);	PM2.5 exposure and deferoxamine (DFO); doses of 10 mg/kg (PM2.5) and 100 mg/kg (DFO) compared with the control group; intranasal exposure twice weekly (15 administrations over 44 days);	not assessed	iron accumulation and the activation of ferritinophagy.	

PAHs—polycyclic aromatic hydrocarbons; PM—particulate matter.

**Table 3:** Health-Based Guidance Values (TDI/RfD) and Regulatory Status for Selected Environmental Compounds.

Compound	Authority/Jurisdiction	Value Type/Exposure Route	Assessment Year	Value	Regulatory Status/Scope	Reference
BPA	EFSA (EU)	Tolerable Daily Intake (TDI)—Oral	2023	0.2 ng/kg bw/day (0.0002 µg/kg bw/day)	Health-Based Guidance Value; re-evaluated and reduced 20,000-fold from the 2015 t-TDI	[114]
BPA	US EPA (USA)	Reference Dose (RfD)—Oral	1988	50 µg/kg bw/day	Health-Based Guidance Value; historical screening level	[115]
BPF	EFSA/ ECHA (EU)	TDI—Oral	2024	No TDI established	Evaluation of alternatives ongoing; under substance evaluation under REACH	[116]
BPP	EFSA (EU)	TDI—Oral	2024	No TDI established	Evaluation of alternatives ongoing	[117]
DBP	EFSA (EU)	Group TDI—Oral	2019	50 µg/kg bw/day	Health-Based Guidance Value (HBGV); group approach (expressed as DEHP equivalents for DBP, BBP, DEHP, DINP)	[118]
DEHP	EFSA (EU)	Group TDI—Oral	2019	50 µg/kg bw/day	Health-Based Guidance Value (HBGV); group approach (expressed as DEHP equivalents for DBP, BBP, DEHP, DINP)	[118]

BPA—bisphenol A; BPF—bisphenol F; BPP—bisphenol P; DBP—dibutyl phthalate; DEHP—di(2-ethylhexyl) phthalate; TDI—tolerable daily intake; RfD—reference dose; EFSA—European Food Safety Authority; US EPA—United States Environmental Protection Agency; EU—European Union; REACH—Registration, Evaluation, Authorisation and Restriction of Chemicals.

**Table 4:** Effect of EDCs on the gut and the liver—data from animal studies.

Reference (Year)	Experimental Model/Study Design	Exposure	Gut Outcomes	Liver Outcomes	Evidence Linking Gut and Liver
Feng et al. (2020) [16]	8 mice per group (control and BPA-treated groups);	BPA 50 μg/kg body weight daily at a concentration of 0.5 mg/kg of food exposure duration of 24 weeks	decreased diversity of intestinal microflora; increased abundance of bacteria from the phylum Proteobacteria, while decreased abundance of Verrucomicrobia. decreased abundance of beneficial *Akkermansia* bacteria; blurred cell boundaries in the anterior part of the small intestine and chaotic distribution of microvilli; disruption of tight junctions between intestinal epithelial cells; decreased production of intestinal structural proteins: ZO-1 and occludin; increased levels of leaky gut markers: LPS DAO and D-lactate;	increased liver weight and a higher ratio of liver weight to total body weight in the animals; irregular morphology, and many of them had small vacuoles; excessive accumulation of lipid droplets; significant increase in TC and TG content in the liver; increased activity of liver enzymes: AST and ALT; increased expression of TLR4 and p-NF-κBp65; increased mRNA expression in the liver; increased serum levels of key inflammatory cytokines: TNF-α, IL-6, IL-1β, and IL-18;	direct gut–liver mediation
Liu et al. (2022) [124]	32 male Sprague Dawley rats animals randomly assigned to 4 groups (*n* = 8 per group) following a 10-day acclimatization period;	BPA; doses of 30 mg/kg, 90 mg/kg, or 270 mg/kg body weight alongside a control group; oral exposure for 30 days;	increased ratio of Firmicutes to Bacteroidetes bacteria (at the highest BPA dose, a 17% decrease in the relative abundance of Bacteroidetes was observed, while the proportion of Firmicutes increased by 17%); decreased abundance of the beneficial bacteria *Prevotella_9 and Ruminococcaceae_UCG-014*; increased abundance of Proteobacteria; decreased acetate, propionate, isobutyrate, valerate, and isovalerate;	increased liver mass and liver quotient (ratio of liver mass to body weight); decreased activity of antioxidant enzymes (e.g., SOD and GSH) and a concomitant increase in MDA levels; congestion of the liver sinusoids, degeneration and necrosis of hepatocytes, and inflammatory cell infiltration; increased expression of the proinflammatory marker IL-1β; significant increase in ALT and AST enzyme levels; lipid profile disturbances (increased TC and LDL-C levels with decreased HDL-C); increased expression of proapoptotic proteins (Bax, cleaved-Caspase3) and decreased concentration of the antiapoptotic protein Bcl; increased liver damage due to decreased concentrations of proteins and genes responsible for mitochondrial biogenesis and stress response (SIRT1, PGC-1α, Nrf1, Nrf2, TFAM);	concurrent gut and liver findings
Wang et al. (2022) [125]	Zebrafish (*Danio rerio*, wild-type AB strain); approximately 120 adult fish per experimental group (3 tanks of approx. 40 individuals); group sizes varied by assay type (e.g., 40 fish/group for liver metabolomics; 90 fish/group for gut microbiome analysis);	BPF; exposure initiated at the early embryonic stage (2 h post-fertilization, 2 hpf) and continued until adulthood concentration groups of 0 μg/L (control and solvent control), 0.5 μg/L, 5.0 μg/L, and 50 μg/L	changes in the composition and diversity of bacteria in the intestines; decrease in the abundance of bacteria from the *Erysipelotrichaceae* and *Rhodobacteraceae* families and the *Gemmobacter* genus; increased abundance of pathogenic Ralstonia bacteria in the highest dose group; intestinal inflammation; significant increase in the amount of acidic and neutral mucus secreted by goblet cells within the intestines;	inhibition of fish growth (reduced body weight and length), while simultaneously causing an abnormal increase in liver weight and liver function index (HSI) in fish exposed to the highest dose (50 μg/L); liver tissue damage; steatosis (the appearance of lipid vacuoles in hepatocytes) was observed in all groups exposed to BPF, the severity of which increased with the dose; development of liver fibrosis was observed in the 5 and 50 μg/L groups; at a concentration of 0.5 μg/L, amino acid metabolism was primarily impaired; at a concentration of 5.0 μg/L, purine metabolism was impaired; at a concentration of 50 μg/L, a collapse of the so-called one-carbon metabolism (folate and methionine cycle) was observed;	concurrent gut and liver findings
Ma et al. (2026) [126]	4-week-old male C57BL/6 mice; divided into 4 experimental groups: control, BPP-treated, antibiotic-treated (ABX), and combined ABX + BPP	BPP; oral gavage dissolved in corn oil at a dose of 30 μg/kg body weight daily; exposure duration of 5 weeks;	increased ratio of Firmicutes to Bacteroidetes bacteria; increased abundance of *Desulfovibrionaceae* bacteria; decreased abundance of *Akkermansia* bacteria; decreased expression of ZO-1, occludin, claudin-4; decreased mucus secretion (decreased Muc2 gene expression); decreased production of sIgA antibodies and antibacterial lectins (Reg3b, Reg3g); macrophage infiltration in the colon; increased expression of proinflammatory proteins and genes (NF-κB, TNF-α, IL-1β, IL-17, IL-23); decreased anti-inflammatory cytokine IL-10	significant increase in endotoxin (LPS) concentration in the blood; increased production of proinflammatory cytokines, including IL-6, TNF-α, and MCP-1; liver enlargement; increased fat droplets in the liver; stimulation of genes responsible for the synthesis and uptake of fatty acids (PPAR-γ and CD36); increased levels of TG and cholesterol (TC and LDL); increased levels of the oxidative damage marker (MDA), and decreased activity of the antioxidant enzyme (SOD); subtle collagen deposition was observed, indicating the beginnings of liver fibrosis; increased levels of liver damage enzymes (AST and ALT) in the blood. histological findings showed degeneration, hepatocellular edema, and inflammatory infiltrates;	direct gut–liver mediation
Xiong et al. (2020) [127]	6-week-old male C57BL/6J mice; divided into 3 main groups of 8 mice each; additional sub-cohorts (*n* = 5 per group) dedicated to microbiome sequencing per group) dedicated to microbiome sequencing	DBP; oral gavage dissolved in corn oil daily dosses of 0 mg/kg (control), 0.1 mg/kg, and 1 mg/kg body weight daily; exposure duration of 10 weeks;	significant decrease in the overall diversity of the gut microbiota (reduction in the Shannon and Simpson indices); increase in the abundance of Firmicutes and Proteobacteria, with a simultaneous decrease in Bacteroidetes and Verrucomicrobia; decrease in the abundance of the beneficial bacteria *Akkermansia*, as well as *Oscillospira* and *Parabacteroides*; decrease in the volume of protective mucus secreted in the intestines; decrease in the expression of genes and proteins forming tight junctions between epithelial cells (occludin and claudin-1); histopathological changes and significantly increased expression of proinflammatory cytokines, including IL-6 and IL-1β, were observed in colonic tissue; influx of LPS in plasma;	increased body weight and liver weight; numerous fat droplets deposited in hepatocytes; activation of the expression of key genes responsible for cholesterol and fatty acid synthesis (including *Srebp-1c*, *Srebp-2*, *Fasn*, *Acc*); increased levels of TG, TC, and LDL in both liver tissue and circulating blood (with a simultaneous decrease in HDL cholesterol); decreased expression of the PPAR-γ receptor; increased production of proinflammatory cytokines in the liver: TNF-α, IL-6, and IL-1β; increased blood enzyme levels—AST, ALT, and, at a lower dose, also ALP;	concurrent gut and liver findings
Li et al. (2025) [128]	3-week-old male Sprague-Dawley rats; total of 20 animals divided after 1 week of acclimatization into 2 equal groups (n=10 per group: control and DEHP-treated);	DEHP; oral gavage dissolved in olive oil at a dose of 3 g/kg body weight daily; short-term exposure dura-tion of 10 consecutive days;	increase in the ratio of Firmicutes to Bacteroidetes bacteria; increase in the number of *Lactobacillus*, *Romboutsia*, and *Alistipes* bacteria, among others; significant decrease in the number of beneficial bacteria of the *Akkermansia* genus (which improve the intestinal barrier), as well as *Bifidobacterium*, unclassified *Muribaculaceae*, and *Oscillibacter* was observed; inhibition of sphingolipid metabolism, as well as disturbances in the metabolism (biosynthesis and degradation) of branched-chain amino acids (valine, leucine, isoleucine); physical damage and shortening of tight junctions in the colon; the occurrence of submucosal edema; significant decrease in the genes and proteins of key barrier components: ZO-1, occludin, and claudin-1; increased levels of LBP (lipopolysaccharide-binding protein))	slow weight gain— significant increase in liver weight and liver function test result was observed; congestion and dilation of the liver sinuses, swelling and shrinkage of hepatocytes, appearance of necrotic foci (necrosis); significant increase in blood levels of marker enzymes: AST and ALT; increased levels of Th1 cell response cytokines (IL-1β, IL-2, TNF-α) and Th2 responses (IL-4, IL-10, IL-13); changes in the expression of as many as 889 genes;	concurrent gut and liver findings

BPA—bisphenol A; BPF—bisphenol F; BPP—bisphenol P; DBP—dibutyl phthalate; DEHP—di(2-ethylhexyl) phthalate; SCFAs—short-chain fatty acids; LPS—lipopolysaccharide; LBP—lipopolysaccharide-binding protein; DAO—diamine oxidase; TC—total cholesterol; TG—triglycerides; LDL-C—low-density lipoprotein cholesterol; HDL-C—high-density lipoprotein cholesterol; AST—aspartate aminotransferase; ALT—alanine aminotransferase; ALP—alkaline phosphatase; SOD—superoxide dismutase; GSH—glutathione; MDA—malondialdehyde; TLR4—Toll-like receptor 4; NF-κB—nuclear factor kappa B; TNF-α—tumor necrosis factor alpha; IL—interleukin; MCP-1—monocyte chemoattractant protein-1; Bax—Bcl-2-associated X protein; Bcl-2—B-cell lymphoma 2 protein; SIRT1—sirtuin 1; PGC-1α—peroxisome proliferator-activated receptor gamma coactivator 1-alpha; Nrf1—nuclear respiratory factor 1; Nrf2—nuclear factor erythroid 2-related factor 2; TFAM—mitochondrial transcription factor A; PPAR-γ—peroxisome proliferator-activated receptor gamma; CD36—cluster of differentiation 36; ZO-1—zonula occludens-1; sIgA—secretory immunoglobulin A; Reg3b—regenerating islet-derived protein 3 beta; Reg3g—regenerating islet-derived protein 3 gamma; Muc2—mucin 2; HSI—hepatosomatic index; ABX—antibiotic treatment; hpf—hours post-fertilization; BCAA—branched-chain amino acids; Fasn—fatty acid synthase; Acc—acetyl-CoA carboxylase; Srebp-1c—sterol regulatory element-binding protein 1c; Srebp-2—sterol regulatory element-binding protein 2.

## Data Availability

No new data were created or analyzed in this study. Data sharing is not applicable to this article.
